# Ecological drivers of avian diversity in a subtropical landscape: Effects of habitat diversity, primary productivity and anthropogenic disturbance

**DOI:** 10.1002/ece3.9166

**Published:** 2022-07-30

**Authors:** Ling‐Ying Shuai, Shu‐Ping Xiao, Yan‐Ping Xie, Xing‐Min Chen, Xiang‐Rong Song, Tian‐Qiao Fan, Yun‐Hua Xie, Wei Liu

**Affiliations:** ^1^ College of Life Sciences Huaibei Normal University Huaibei China; ^2^ Mingxi Forestry Bureau Mingxi China; ^3^ College of Life Sciences Henan Normal University Xinxiang China; ^4^ Northwest A&F University Yangling China

**Keywords:** anthropogenic disturbance, birdwatching, functional diversity, habitat diversity, phylogenetic diversity, species richness

## Abstract

Understanding the roles of ecological drivers in shaping biodiversity is fundamental for conservation practice. In this study, we explored the effects of elevation, conservation status, primary productivity, habitat diversity and anthropogenic disturbance (represented by human population density and birding history) on taxonomic, phylogenetic and functional avian diversity in a subtropical landscape in southeastern China. We conducted bird surveys using 1‐km transects across a total of 30 sites, of which 10 sites were located within a natural reserve. Metrics of functional diversity were calculated based on six functional traits (body mass, clutch size, dispersal ratio, sociality, diet and foraging stratum). We built simultaneous autoregression models to assess the association between the ecological factors and diversity of the local avian communities. Local avian diversity generally increased with increasing habitat diversity, human population density and primary productivity. We also detected phylogenetic and functional clustering in these communities, suggesting that the avian assemblages were structured mainly by environmental filtering, rather than interspecific competition. Compared with sites outside the natural reserve, sites within the natural reserve had relatively lower avian diversity but a higher level of phylogenetic heterogeneity.

## Graphical abstract

Across a subtropical landscape in China, we found both phylogenetic and functional clustering in avian communities, suggesting that the local avian assemblages were structured mainly by environmental filtering, rather than niche partitioning. The relationship between anthropogenic disturbance and avian diversity is complex, as we found both a positive association between human population density and avian diversity, and a significantly negative relationship between birding history and species richness.

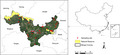

## 1 INTRODUCTION

Understanding the roles of ecological drivers in shaping biodiversity patterns is a fundamental task for ecologists and conservation biologists (Chesson, 2000; Gaston, 2000). In this regard, several fundamental theories have been proposed. Among them, the ‘productivity hypothesis’ (Hurlbert & Haskell, 2003) and the ‘habitat heterogeneity hypothesis’ (Terborgh, 1977) may be most well‐known. The former states that higher primary productivity can sustain more species via trophic cascades (Hurlbert & Haskell, 2003), while the latter emphasizes the role of habitat diversity and niche partitioning (Guégan et al., 1998; Tews et al., 2004). To test these hypotheses, numerous field studies on taxonomic diversity (namely species richness) have been conducted and empirical support has been accumulated (Bailey et al., 2004; Ben‐Hur & Kadmon, 2020; Hawkins et al., 2003). However, taxonomic diversity generally assumes that all species are equivalent, ignoring evolutionary and ecological differences between species. As a result, studies on biodiversity have recently been extended to phylogenetic diversity and functional diversity.

For a given community, phylogenetic diversity reflects the diversity of lineages (Faith, 1992), while functional diversity measures the range and diversity of traits linked with functions and life history (Petchey et al., 2007). Adopting a multi‐faceted diversity framework combining phylogenetic and functional measures would promote our understanding of community assembly rules and interactions between diversity and ecological processes (Mouchet et al., 2010). For instance, if environmental filtering is the dominant assembly process, phylogenetic or functional clustering should be expected, i.e., the community is mainly composed of ecologically or evolutionarily similar species (Mouchet et al., 2010); however, if interspecific competition is more important, we should observe phylogenetic or functional overdispersion, as the results of limiting similarity (MacArthur & Levins, 1967; Mouchet et al., 2010).

Among the many factors associated with biodiversity patterns, anthropogenic disturbance has received considerable attention (Asefa et al., 2017; Eggleton et al., 2002; Gorczynski et al., 2021; Mishra et al., 2004; Zhu et al., 2007). In many cases, anthropogenic disturbance is linked with detrimental processes causing extinction and diversity loss, such as deforestation (Horgan, 2005), habitat fragmentation (Wilson et al., 2016), biological invasions (Li et al., 2016) and overexploitation (Chen et al., 2019). In this scenario, human population density is often used as an important surrogate for anthropogenic disturbance. However, the relationship between anthropogenic disturbance and diversity may also be positive (Shuai et al., 2021), as disturbance can take many forms and some types of disturbance may even promote diversity (Heim et al., 2022; Tocco et al., 2020). For example, cultivation may promote habitat diversity by turning some forests into crop fields, and fields themselves also provide important food resources for many species. A survey in a tropical agricultural landscape suggests that crop heterogeneity can help to promote avian diversity (Lee & Goodale, 2018). Moreover, some types of environment‐friendly tourism have been proposed as an important solution for protecting biodiversity. Birdwatching tourism, for instance, has been widespread throughout the world in recent decades (Ma et al., 2013). Since the last decade, birdwatching tourism has also been launched in some natural reserves in China. Birdwatching incentivizes biodiversity conservation by involving local communities and tourists in the protection of interesting birding sites (Cooper et al., 2015). In this sense, birdwatching tourism has been considered an important force for conservation (Ma et al., 2013). However, the actual effect of birdwatching tourism on avian diversity remains understudied (Sekercioglu, 2002).

In this study, we explored the effects of productivity, habitat diversity, and anthropogenic disturbance on taxonomic, phylogenetic and functional avian diversity across a subtropical city in southeastern China. We focused on two questions: (i) What are the main ecological drivers of avian diversity on a local scale? We also predicted that avian diversity should increase with primary productivity and habitat diversity, in accordance with the ‘productivity hypothesis’ and the ‘habitat heterogeneity hypothesis’. (ii) Which process is more likely to dominate the local bird community assembly, environmental filtering or interspecific competition?

## 2 METHODS

### 2.1 Study area

This study was conducted in a subtropical landscape across the whole Mingxi County in Fujian Province, Southeastern China, ranging between 116°47′–117°35′E and 26°08′–26°39′N. This landscape is mainly broadleaved evergreen forest, which cover an area of 1730 km^2^. The climate is warm and moist, with mean annual rainfall of 1800 mm and mean annual temperature of 18°C.

**FIGURE 1:**
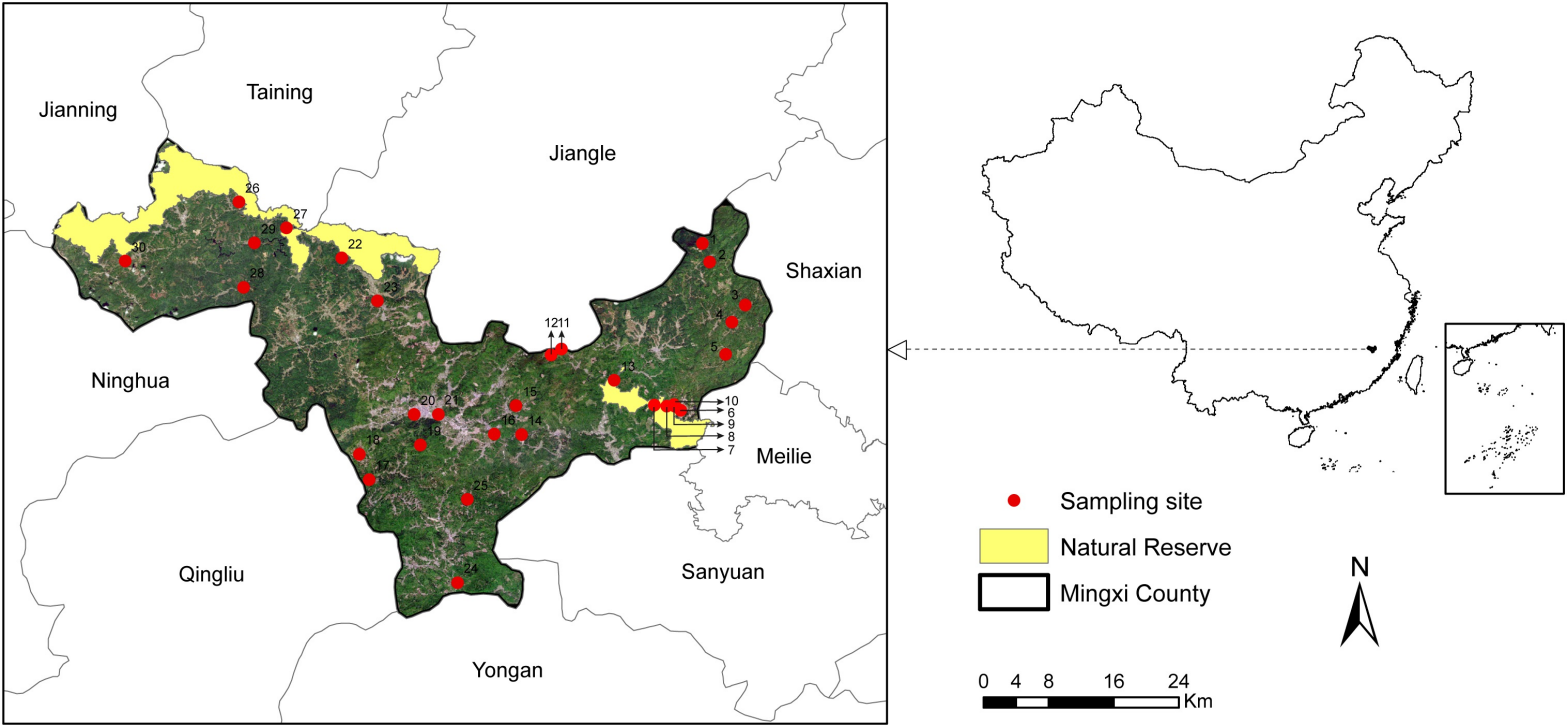
Study area and locations of sampling sites. Yellow area shows the range of the Junzifeng national natural reserve.

We selected 30 sampling sites across the landscape, with a minimum interval of 1 km between sites (Figure 1). Each site had a circular shape, with a radius of 1 km. Among these sites, 10 were located in the buffer area of Junzifeng National Nature Reserve, which was founded in 1995 and has an area of 180.61 km^2^, or about 10.4% of the total area of Mingxi County. Since Mingxi harbors a high avian diversity (a total of 320 species recorded so far) and many flagship avian species, birdwatching tourism has been widespread throughout the city in the last decade (Huang & Xiao, 2016). In our study, half of the sites (15 sites) had a birding history (i.e., number of years since birdwatching tourism was started in a site) longer than 0, among which 10 sites had a birding history of over 4 years.

### 2.2 Bird survey

From 2019 to 2020, bird surveys were conducted four times per year (March, June, September and December) at all the sites, using the standard line‐transect method. In each site, we established a 1‐km transect passing through the major habitat types (e.g., broadleaved evergreen forest, cropland and bamboo forest) found within the site. In each survey, two experienced observers walked at a speed of 1.5 km/h along each transect and recorded all the bird species seen or heard within 100 m on each side of the transect, as well as its foraging stratum (roughly categorized as ground, understorey, middle, canopy and air) when possible. All the surveys were carried out on rainless and windless days during the periods with relatively high avian activity, i.e., between 30 min after dawn to 11:00 h or between 15:00 h to 30 min before sunset (Wang et al., 2010). The abundance data of each avian species per site can be found in Table S1. We also checked the conservation status of the recorded species according to the IUCN red list (IUCN, 2017).

### 2.3 Functional traits

To calculate functional diversity of bird communities, a total of six traits were selected (Table S2), including three continuous traits (mean body weight, mean clutch size, and mean dispersal ratio) and three categorical traits (sociality, diet, and foraging stratum). Body weight is usually associated with energy demands and ecological impacts of a species, and has been viewed as one of the most fundamental functional traits (Ding et al., 2013). As a measure to evaluate a species' mobility, dispersal ratio for each species was calculated by dividing its mean wing length by the cube root of its mean body weight (Wang et al., 2015). Sociality was defined as either social (either in small or large groups) or solitary (Wang et al., 2018). Diet included three non‐exclusive binary attributes (three food types): plants, invertebrates and vertebrates. A species' diet can thus include one, two or three food types (Li et al., 2019). Data on foraging stratum was mainly based on our records during the field surveys, and we also used EltonTraits (Wilman et al., 2014) as a supplementary reference when reliable records were unavailable. We collected the other trait data from two recent publications (Liu & Chen, 2021; Wang et al., 2018), as well as a global trait database for amniotes (Myhrvold et al., 2015).

### 2.4 Diversity metrics

To reduce the effects of inter‐annual variations, we used accumulated abundance of each species and species richness through the whole sampling period in this study (Zhang et al., 2020). To evaluate taxonomic diversity, species richness was represented by the observed number of species within each site accumulated throughout the whole survey. To take into consideration the effect of sample size, we also rarefied species richness to the same number of individuals using the package ‘vegan’ (Oksanen, 2017).

To calculate phylogenetic diversity, we obtained 2000 phylogenetic trees including all the bird species recorded in our survey from BirdTree (http://birdtree.org), using the “Ericson” backbone (Jetz et al., 2012). These trees were then summarized using SumTrees to generate a 50% majority rule concensus tree. Using the concensus tree, mean pairwise phylogenetic distance (hereafter PhyloMPD) and mean nearest phylogenetic distance (hereafter PhyloMNTD) were calculated. As a surrogate for total divergence of the community, PhyloMPD was calculated by averaging all the pairwise phylogenetic distances (i.e., branch lengths on the phylogenetic tree) among species co‐occurring in a community. PhyloMNTD was calculated by averaging the minimum phylogenetic distance between species pairs. To some extent, PhyloMPD and PhyloMNTD are complementary measurements, as PhyloMPD is more sensitive to the signal of over‐dispersion (Mazel et al., 2016), while PhyloMNTD provides information on the tips of the phylogeny.

Similar to PhyloMPD and PhyloMNTD, we also calculated MPD and MNTD for functional traits (hereafter FunctMPD and FunctMNTD). As we had both continuous and categorical trait data, we used the Gower's distance to calculate the pairwise inter‐specific functional distance matrix (Gower, 1966). We then generated a dendrogram using the unweighted pair group method with arithmetic mean (UPGMA; Swenson, 2014). Based on this functional dendrogram, FunctMPD was calculated by averaging all pairwise functional distances (branch lengths on the functional dendrogram) among co‐occurring species within a site, and FunctMNTD was calculated by averaging the functional distance between nearest neighbors (Li et al., 2019).

We further calculated the standard effect sizes for both MPD and MNTD as follows:

$$\mathrm{ses}.\mathrm{MPD}=\left({\mathrm{MPD}}_{\text{null}}-{\mathrm{MPD}}_{\mathrm{obs}}\right)/{\mathrm{SD}}_{\text{null}}$$

$$\mathrm{ses}.\text{MNTD}=\left({\text{MNTD}}_{\text{null}}-{\text{MNTD}}_{\mathrm{obs}}\right)/{\mathrm{SD}}_{\text{null}}$$

where ses.MPD/ses.MNTD refers to standard effect size for either MPD or MNTD, MPD_null_/MNTD_null_ is the mean value of MPD or MNTD from the 999 randomly simulated communities, and MPD_obs_/MNTD_obs_ is the observed value of MPD or MNTD. For these two indicators, a value <0 suggests phylogenetic or functional clustering, while a value >0 suggests overdispersion. MPD, MNTD, ses.MPD and ses.MNTD were calculated using the package ‘picante’ (Kembel, 2010). Values of all the diversity indices were listed in Table S3.

### 2.5 Ecological factors

We collected information on a total of five habitat factors: elevation, conservation status (whether a site was located in the natural reserve or not), normalized difference vegetation index (NDVI), habitat diversity (represented by the Shannon‐Wiener index) and area of largest forest patch (Table S4). Elevation of each site was recorded using a hand‐held GPS (UniStrong A5, Beijing). As a surrogate for primary productivity, NDVI of each site was obtained using the grid data (with a 100 × 100 m resolution) provided by the Data Center for Resources and Environmental Sciences, Chinese Academy of Sciences (RESDC, http://www.resdc.cn).

To investigate the area of each habitat type, we downloaded a satellite image (LC81200422018301LGN00, Landsat 8 thematic mapper, March 2018) from the Geospatial Data Cloud (http://www.gscloud.cn/sources). This image had a spatial resolution of 30 × 30 m and covered the entire study area. Landscape interpretation was performed in ERDAS IMAGINE 9.2 (Zeng et al., 2012). We classified the habitats into six categories: river, road, buildings, broadleaved forest, bamboo forest and field. For each site, we calculated the area (in hectare) of each habitat type in ArcGIS (version 10.2.2). Based on the area of each habitat type, we calculated the Shannon‐Wiener index for each site using the package ‘vegan’(Oksanen, 2017), and recorded the area of the largest forest patch (only considering broad‐leaved forest) found within each site.

Two attributes were used to reflect anthropogenic disturbance. First, we adopted AcrGIS to obtain the population density (in the year 2015) within each site, based on the grid data (with a 1 × 1 km resolution) provided by RESDC. Second, to evaluate the potential effect of birdwatching tourisms on avian community, we calculated the duration of birdwatching tourism (hereafter, birding history) in each site, i.e., the year we finished the survey (2021) minus the year when birdwatching tourism started in each site. We obtained this information from Mingxi Forestry Bureau, which directed and monitored birdwatching tourism in the whole county. The birding history ranged from 0 to 7 years among the 30 sites, and the sites within the natural reserve had significantly longer birding histories (Mann–Whitney test: *p* = .031) than those outside the natural reserve.

### 2.6 Statistical analyses

First, we explored the effects of ecological factors on each of the six diversity metrics (species richness, rarefied richness, sesPhyloMPD, sesPhyloMNTD, sesFunctMPD and sesFunctMNTD). All the seven ecological factors (elevation, conservation status, NDVI, habitat diversity, area of largest forest patch, human population density and birding history) were included as explanatory factors. Considering the potential effects of spatial autocorrelation, we built simultaneous autoregression (SAR) models using the package ‘spatialreg’ (Bivand et al., 2021). Because our sample size was relatively small (30 sites), only main effects were considered. We performed model selection based on corrected Akaike information criterion (AIC_c_). As no single best model can be achieved (due to the small differences in AIC_c_), we then adopted conditional model averaging on the whole model set to achieve an ‘averaged’ model. Model selection and model averaging were conducted using the package ‘MuMIn’(Bartoń, 2016). To evaluate the potential effects of multicollinearity, we adopted the function ‘*vif*’ from the package ‘*car*’ to calculate the variance inflation factor for each explanatory factor (Fox & Weisberg, 2019). VIFs were relatively small (elevation: 1.35; conservation status: 1.62; NDVI: 2.26; habitat diversity: 3.41; area of largest forest patch: 3.08; human population density: 1.16; birding history: 1.57).

Second, to explore whether phylogenetic or functional clustering or overdispersion occurs within a community, we adopted t tests to explore whether ses.MPD and ses.MNTD were significantly different from 0, as should be expected by chance. All the statistical work were performed in R 3.5.3 (R core team, 2019).

## 3 RESULTS

A total of 175 avian species (13,306 records) were recorded during the 2‐year field survey (Table S1), among which 95 species (54.28%) were passerines. The 10 most abundant species were all passerines: White‐rumped Munia (*Lonchura striata*), Scaly‐breasted Munia (*Lonchura punctulata*), Crested Myna (*Acridotheres cristatellus*), Red‐rumped Swallow (*Cecropis daurica*), Light‐vented Bulbul (*Pycnonotus sinensis*), Collared Finchbill (*Spizixos semitorques*), Barn swallow (*Hirundo rustica*), Red‐billed Blue Magpie (*Urocissa erythroryncha*), Eurasion Tree Sparrow (*Passer montanus*) and Black Bulbul (*Hypsipetes leucocephalus*), representing 5753 records or 43.23% of the total records. We recorded three Chinese endemic species: White‐necklaced Partridge (*Arborophila gingica*), Elliot's Pheasant (*Syrmaticus ellioti*) and Chinese Bamboo Patridge (*Bambusicola thoracicus*). We also recorded two threatened species: Scaly‐sided Merganser (*Mergus squamatus*; endangered) and Rustic Bunting (*Emberiza rustica*; vulnerable) according to the IUCN Red List. Protected and unprotected sites showed similar levels of habitat diversity (*t* test: *t* = 0.094, *df* = 28, *p* = .93).

**FIGURE 2:**
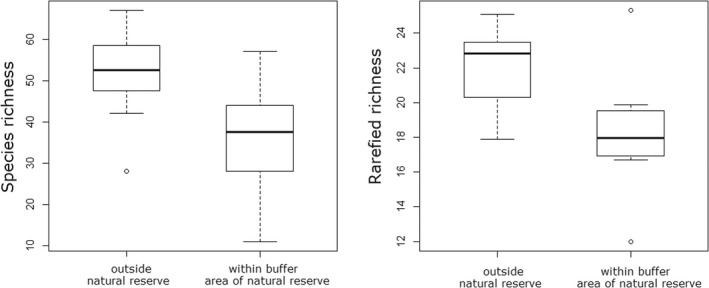
Comparison between sites within the natural reserve and outside the natural reserve on species richness and rarefied richness.

According to model averaging, sites within the natural reserve had lower species richness (Figure 2), and species richness increased with increasing habitat diversity, increasing NDVI, and increasing human population density (Table 1). Rarefied richness was also lower in the natural reserve (Figure 2), and positively related with habitat diversity and area of largest forest patch (Table 1).

**TABLE 1:** Results of model averaging (conditional average) of simultaneous autoregression (SAR) models on species richness and rarefied richness. Significant correlations were marked in bold (*p* < .05).

Dependent variable	Independent variable	Coefficients	SE	*Z* value	*p*
Species richness	Intercept	−34.10	49.54	0.69	.49
	Elevation	0.0097	0.014	0.71	.48
	**Conservation status**	−12.05	3.50	3.44	<.001
	**Habitat diversity**	31.02	11.25	2.76	.0058
	**NDVI**	65.24	25.26	2.55	.011
	Area of largest forest patch	0.047	0.065	0.73	.47
	**Population density**	0.23	0.11	2.14	.032
	Birding history	−0.13	0.62	0.21	.83
Rarefied richness	Intercept	10.47	10.40	1.006	.31
	Elevation	−0.00025	0.0025	0.10	.92
	**Conservation status**	−3.10	1.02	3.03	.0025
	**Habitat diversity**	7.51	3.35	2.24	.025
	NDVI	9.16	7.21	1.27	.20
	**Area of largest forest patch**	0.039	0.018	2.12	.034
	Population density	0.048	0.035	1.37	.17
	Birding history	−0.25	0.19	1.33	.19

**FIGURE 3:**
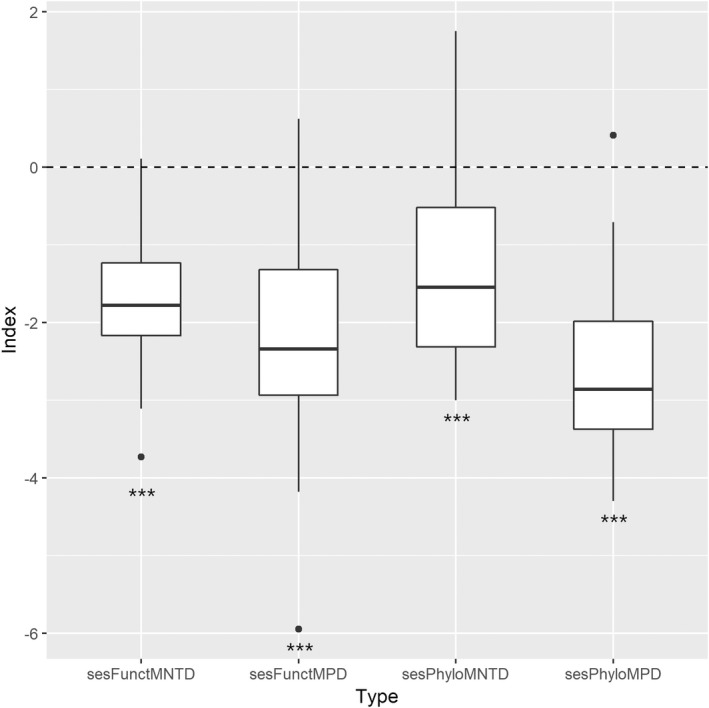
Boxplots of standardized effect sizes of functional mean pairwise distance (sesFunctMPD), functional mean nearest taxon distance (sesFunctMNTD), phylogenetic mean pairwise distance (sesPhyloMPD) and phylogenetic mean nearest taxon distance (sesPhyloMNTD). Asterisk indicates significantly different to 0.

**FIGURE 4:**
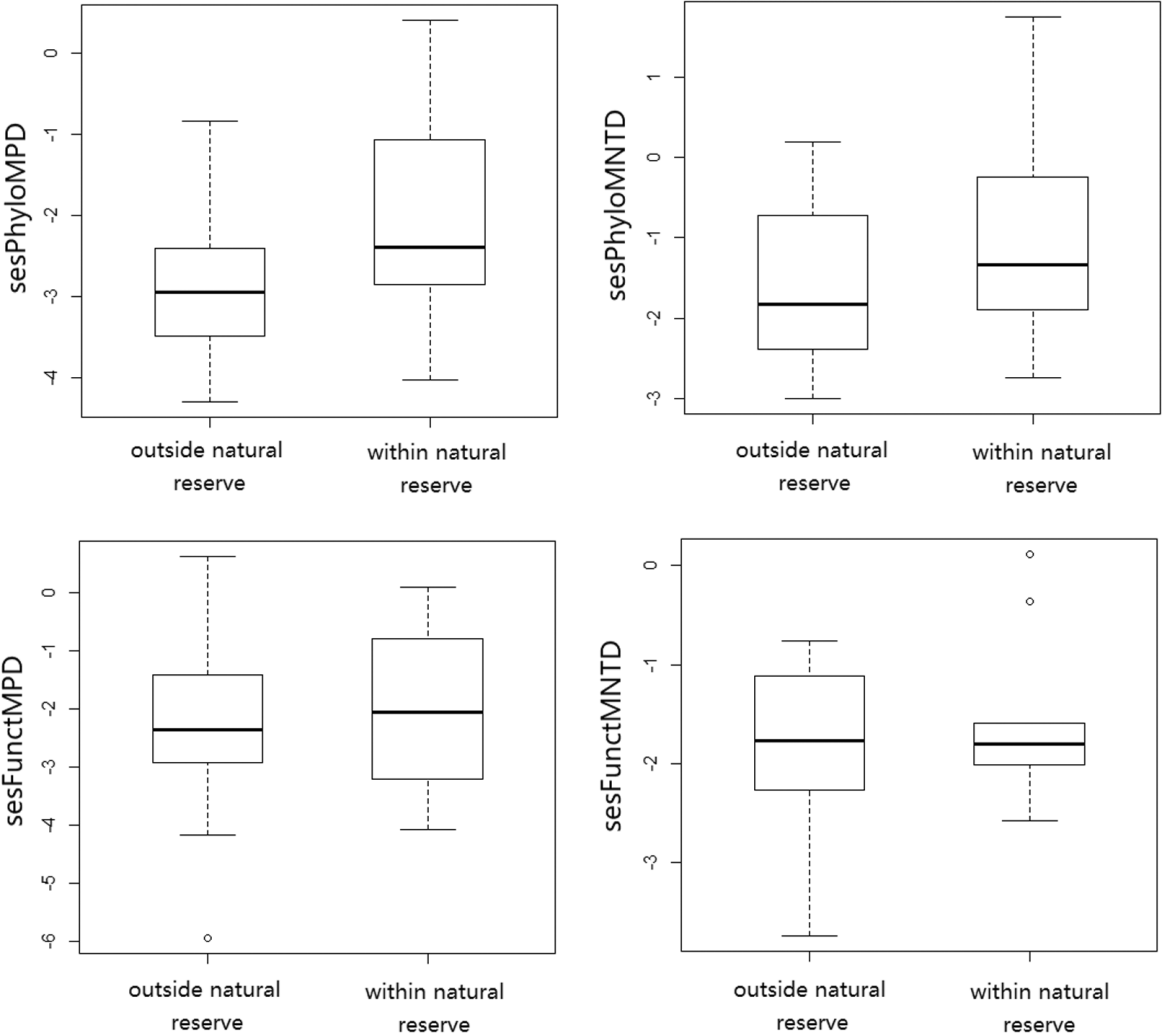
Comparison between sites within the natural reserve and outside the natural reserve on standardized effects of phylogenetic mean pairwise distance (sesPhyloMPD), standardized effects of phylogenetic mean nearest taxon distance (sesPhyloMNTD), standardized effects of functional mean pairwise distance (sesFunctMPD), standardized effects of functional mean nearest taxon distance (sesFunctMNTD).

In terms of ses.PhyloMPD, phylogenetic clustering was detected in 23 sites, while no significant overdispersion or clustering was found in the other 7 sites. Average ses.PhyloMPD was significantly lower than 0 (*t* = −11.96, *df* = 29, *p* < .001), suggesting an overall phylogenetic clustering in this region (Figure 3). Tests on ses.PhyloMNTD generated similar results (*t* = −6.75, *df* = 29, *p* < .001; Figure 3). Sites within the natural reserve had significantly higher ses.PhyloMPD and ses.PhyloMNTD than sites outside the natural reserve (Table 2; Figure 4). According to ses.FunctMPD, functional clustering was also detected in 19 sites, resulting in an overall functional clustering (*t* = −8.65, *df* = 29, *p* < .001; Figure 3). Similarly, ses.FunctMNTD was significantly lower than 0 (*t* = −11.76, *df* = 29, *p* < .001; Figure 3), supporting the idea that the avian communities tended to be functionally clustered across the sites. No significant relationship between explanatory factors and the two functional diversity metrics was detected (Table 3).

**TABLE 2:** Results of model averaging (conditional average) of simultaneous autoregression (SAR) models on sesPhyloMPD (standardized effect size of phylogenetic mean pairwise distance) and sesPhyloMNTD (standardized effect size of phylogenetic mean nearest taxon distance).

Dependent variable	Independent variable	Coefficients	SE	*Z* value	*p*
sesPhyloMPD	Intercept	−2.44	1.64	1.49	.14
	Elevation	−0.0012	0.0011	1.12	.26
	**Conservation status**	0.85	0.42	2.04	.042
	Habitat diversity	−0.076	1.31	0.058	.95
	NDVI	0.43	2.99	0.14	.89
	Area of largest forest patch	−0.0037	0.0061	0.61	.54
	Population density	−0.0076	0.017	0.44	.66
	Birding history	0.064	0.085	0.76	.45
sesPhyloMNTD	Intercept	−2.55	2.56	1.00	.32
	Elevation	−0.00061	0.00075	0.82	.41
	**Conservation status**	0.90	0.35	2.56	.010
	Habitat diversity	−1.26	1.21	1.04	.30
	NDVI	3.42	2.80	1.22	.22
	Area of largest forest patch	0.0079	0.0056	1.42	.16
	Population density	0.0076	0.016	0.46	.64
	Birding history	0.11	0.085	1.23	.22

**TABLE 3:** Results of model averaging (conditional average) of simultaneous autoregression (SAR) models on sesFunctMPD (standardized effect size of functional mean pairwise distance) and sesFunctMNTD (standardized effect size of functional mean nearest taxon distance).

Dependent variable	Independent variable	Coefficients	SE	*Z* value	*p*
sesFunctMPD	Intercept	−1.96	3.49	0.56	.57
	Elevation	−0.00037	0.0011	0.34	.74
	Conservation status	0.57	0.51	1.12	.26
	Habitat diversity	2.18	1.33	1.64	.10
	NDVI	−4.18	3.61	1.16	.25
	Area of largest forest patch	−0.011	0.0070	1.50	.13
	Population density	0.017	0.020	0.84	.40
	Birding history	−0.024	0.11	0.23	.82
sesFunctMNTD	Intercept	−1.46	1.29	1.13	.26
	Elevation	0.00040	0.00056	0.71	.48
	Conservation status	0.30	0.28	1.08	.28
	Habitat diversity	−0.80	0.80	1.00	.32
	NDVI	−0.60	2.10	0.29	.77
	Area of largest forest patch	−0.00080	0.0044	0.18	.86
	Population density	−0.00081	0.012	0.066	.95
	Birding history	0.0099	0.058	0.17	.87

## 4 DISCUSSION

In this study, we tested for the effects of elevation, conservation status, primary productivity, habitat diversity, and anthropogenic disturbance on avian diversity across a subtropical city in southeastern China. In terms of taxonomic diversity, conservation status and habitat diversity were the two most consistently important factors in determining avian diversity across the study area. In consistent with some previous studies (Guégan et al., 1998), our results supported both the productivity hypothesis and the habitat heterogeneity hypothesis, as bird species richness increased with increasing NDVI (a surrogate for primary productivity) and habitat diversity.

We also found a positive relationship between human population density and species richness, suggesting that the effects of anthropogenic disturbance on biodiversity are often multifaceted. Several factors may have contributed to this pattern. First, possibly due to long‐term monitoring, education and broadcasting, citizens (especially villagers) in Mingxi City generally possess a pretty good level of awareness of protection. As more and more villagers took part in managing birdwatching tourism, they became more willing to make efforts to protect bird species and reduce their environmental impact. Second, the average population density in the study area was relatively low (62.06 persons/km^2^, while the average population density in Fujian Province is 335 persons/km^2^), suggesting that the negative effect of population on avian diversity may have been small, if any. Finally, in our study area, higher population density is often associated with long‐term cultivation, resulting in partial conversion of forests into crop fields. Such a change in land use may have promoted habitat diversity and provided important food resources to many bird species. The positive effects of wildlife‐friendly agricultural practice on avian diversity have been documented in some previous studies (Cannon et al., 2019; Lee & Goodale, 2018; Sreekar et al., 2021).

Counterintuitively, we found that sites in the natural reserve harbor fewer bird species than outside the reserve. A possible reason is that the sites outside the reserve have higher habitat diversity, due to long‐term cultivation. However, this is not the case in our study, as we detected no significant difference in habitat diversity between sites within and outside the natural reserve. Another reason is related to birdwatching tourism. It should be noted that sites in the natural reserve had significant longer birding history than outside the reserve. Although birdwatching is often viewed as an environment‐friendly type of tourism, the actions of observers and photographers may still cause negative effects on birds and environment, such as disturbing birds, increased nest predation, and visitor‐caused pollution (Sekercioglu, 2002; Slater et al., 2019). As a famous Chinese endemic species, Cabot's tragopan (*Tragopan caboti*) was not detected during this survey, although it has been acting as a flagship species in Mingxi and attracted many photographers to visit these sites. However, results of model averaging suggest no significant relationship between birding history and species richness. We think a plausible explanation for the relatively higher species richness of the sites outside the natural reserve is that these sites often possess some croplands, which may provide important food for some bird species and thus change their distribution, at least in some seasons. Further investigations are required to test this mechanism.

In general, both phylogenetic and functional clustering were detected in the avian communities, suggesting that environmental filtering, rather than limiting similarity, should be the dominant assembly process (Li et al., 2019). It is suggested that environmental filtering and limiting similarity take effects at different spatial scales (Cardillo et al., 2008). Based on abiotic factors (such as climate), environmental filtering may be dominant at relatively large scales, while limiting similarity based on biological interaction may be more important at smaller scales. In our study, however, phylogenetic and functional clustering was detected at local scales. Our results were similar to some previous studies, where phylogenetic or functional clustering at local scales was also found in waterbird communities (Li et al., 2019) or Neotropical forest bird communities (Gomez et al., 2010). In summary, these results suggest that the process of environmental filtering may also be prominent in bird communities even at small scales. We think a plausible explanation for this unexpected pattern is related to some unique traits of birds. Niche partitioning and limiting similarity is mainly associated with stable coexistence among species (Chesson, 2000), which means that each species tends to recover when rare. This may not be the case for many bird communities, however, as birds are highly migratory and the composition of avian communities is often variable between seasons or years, which may greatly reduce the chances of competitive exclusion. In other words, instable or temporary coexistence may be more important in some bird communities. In this scenario, biogeographic processes of migration and the spatial dynamics of meta‐communities may be more important in shaping the structure of local communities (Chesson, 2000; Thuiller et al., 2015).

Finally, it should be noted that although harboring lower species richness, sites within buffer area of the natural reserve still had higher phylogenetic heterogeneity than sites outside the natural reserve. A recent study on a tropical island suggests that economic development and changes in land use may cause increased phylogenetic clustering (Pganani‐Nunez et al., 2022). Our results support this idea, as sites with less economic development (i.e., sites within the natural reserve) were associated with less phylogenetic clustering.

## 5 CONCLUSIONS

In this study, we assessed the roles of productivity, habitat diversity and anthropogenic disturbance in shaping avian diversity across a subtropical landscape, as well as the assembly rule of avian assemblages. In general, models on taxonomic diversity (represented by species richness) support both the productivity hypothesis and the habitat heterogeneity hypothesis. According to the detected phylogenetic and functional clustering, the local avian communities in this study should be mainly shaped by environmental filtering, rather than niche partitioning. There was a positive relationship between human population density and avian diversity. In order to maintain avian diversity across this landscape, it would be important to pay attention to sites with high habitat diversity, as well as potential disturbances on avian communities, especially in sites within the natural reserve.

## AUTHOR CONTRIBUTIONS

**Lingying Shuai:** Conceptualization (equal); formal analysis (lead); writing – original draft (lead). **Shu‐Ping Xiao:** Conceptualization (equal); funding acquisition (lead); investigation (lead). **Yan‐Ping Xie:** Investigation (equal); methodology (equal). **Xing‐Min Chen:** Investigation (equal); methodology (equal). **Xiang‐Rong Song:** Investigation (equal). **Tian‐Qiao Fan:** Investigation (equal). **Yun‐Hua Xie:** Investigation (equal). **Wei Liu:** Conceptualization (equal); project administration (lead); writing – review and editing (lead).

## FUNDING INFORMATION

This work was supported by the Mingxi Forestry Bureau project ‘Extensive bird survey across the Mingxi County’.

## CONFLICT OF INTEREST

The authors declare that they have no conflict of interest.

## Supporting information


**Appendix S1** Supporting Information

**Appendix S2** Supporting Information

**Appendix S3** Supporting Information

**Appendix S4** Supporting Information

## Data Availability

The datasets supporting the conclusions of this article will be archived in Dryad upon acceptance. Dryad doi: https://doi.org/10.5061/dryad.gxd2547q1
