# Supplementary material for: Ecological drivers of avian diversity in a subtropical landscape: Effects of habitat diversity, primary productivity and anthropogenic disturbance
Source: Ecol Evol. 2022 Jul 30;12(8):e9166. doi: 10.1002/ece3.9166 (PMC9338441; doi:10.1002/ece3.9166)
Supplement: Supplementary file 1 — Appendix S1 Supporting Information [file ECE3-12-e9166-s001.pdf]

Appendix 1 Abundance data of avian species

Site No.	*Aix galericulata*	*Anas zonorhyncha*	*Mergus squamatus*	*Arborophila gingica*	*Bambusicola thoracicus*	*Lophura nycthemera*	*Syrmaticus ellioti*	*Phasianus colchicus*	*Tachybaptus ruficollis*	*Ixobrychus sinensis*	*Ixobrychus cinnamomeus*	*Nycticorax nycticorax*
1	0	0	0	0	4	15	0	0	0	0	0	0
2	0	0	0	0	12	1	0	5	0	0	1	0
3	0	0	0	0	7	1	0	2	0	0	6	0
4	0	2	0	0	4	0	0	8	0	1	3	0
5	0	0	0	0	2	8	0	4	0	0	3	0
6	0	0	0	0	1	0	0	9	0	0	2	2
7	0	0	0	0	0	5	0	0	0	0	1	0
8	0	0	0	0	0	0	2	0	1	0	0	0
9	0	0	0	0	0	4	0	2	9	0	0	0
10	0	0	0	0	0	4	6	0	0	0	2	0
11	0	1	0	0	0	19	1	3	0	2	0	0
12	0	0	0	0	4	26	0	4	0	0	0	0
13	0	2	0	0	0	2	1	2	0	0	0	0
14	0	0	0	0	0	0	0	3	0	0	0	0
15	0	0	0	0	0	0	0	14	0	0	0	1
16	0	0	0	0	0	0	0	5	0	0	4	2
17	2	0	0	0	3	0	0	0	0	0	0	0
18	7	0	0	0	1	1	0	8	9	0	0	0
19	0	0	0	2	0	0	1	0	0	0	0	0
20	0	0	0	0	0	0	0	1	5	0	0	1
21	0	0	0	0	1	0	0	0	9	0	1	10
22	20	20	8	2	5	13	0	1	13	0	0	7
23	0	18	0	0	9	0	0	0	0	0	0	0
24	0	12	0	0	0	3	0	0	26	0	0	4
25	0	0	0	0	0	27	0	2	0	0	2	1
26	0	17	0	0	0	0	0	4	0	0	2	0
27	10	6	0	0	0	0	0	0	1	0	0	0
28	19	0	0	1	0	2	0	3	2	0	1	10
29	16	9	0	0	2	30	0	0	0	0	0	0
30	0	0	0	0	1	0	0	0	0	0	2	0

Appendix 1

Site No.	*Butorides striata*	*Ardeola bacchus*	*Bubulcus coromandus*	*Ardea cinerea*	*Ardea alba*	*Ardea intermedia*	*Egretta garzetta*	*Phalacrocorax carbo*	*Elanus caeruleus*	*Pernis ptilorhynchus*	*Aviceda leuphotes*	*Spilornis cheela*	*Ictinaetus malayensis*
1	0	0	0	0	0	0	0	0	0	0	0	3	0
2	0	2	5	0	1	0	7	0	0	0	1	2	3
3	0	1	0	0	0	0	8	0	0	0	2	2	2
4	0	0	6	0	0	0	5	0	0	0	0	0	3
5	0	4	0	0	2	0	1	0	0	0	0	0	1
6	0	1	0	0	0	0	2	0	0	0	2	0	0
7	0	0	0	0	0	0	4	0	0	0	0	0	0
8	0	0	0	0	0	0	0	0	0	0	0	0	0
9	0	1	0	0	0	0	2	0	0	0	0	0	0
10	0	0	0	0	0	0	0	0	0	0	0	0	0
11	0	4	12	0	0	0	6	0	0	0	0	0	0
12	0	1	0	0	0	0	3	0	0	0	0	0	0
13	0	0	8	0	0	0	5	0	0	0	0	0	0
14	0	9	8	0	0	0	9	0	0	0	0	0	0
15	0	0	0	0	1	0	3	0	0	0	0	0	0
16	0	5	7	0	0	0	4	0	0	0	0	0	0
17	0	3	0	0	0	0	1	0	0	0	0	0	0
18	0	2	10	2	0	0	15	0	0	0	0	0	0
19	0	0	0	0	0	0	0	0	0	2	0	0	0
20	0	8	1	0	1	0	4	0	0	0	0	0	0
21	0	12	2	0	0	4	16	0	2	0	0	0	0
22	1	1	0	0	0	0	5	10	0	0	0	0	1
23	0	5	6	0	0	0	28	0	1	0	0	0	0
24	0	5	0	0	0	0	3	0	0	0	0	1	0
25	0	3	6	0	0	0	2	0	0	0	0	0	0
26	0	0	0	0	0	0	2	0	0	0	0	0	0
27	2	4	0	0	0	0	3	0	0	0	0	0	1
28	1	2	0	0	0	0	5	0	0	0	0	0	0
29	0	1	1	0	0	0	10	0	0	0	0	0	0
30	0	2	0	0	0	0	0	0	0	0	0	0	0

Appendix 1

Site No.	*Accipiter trivirgatus*	*Accipiter soloensis*	*Accipiter virgatus*	*Circus spilonotus*	*Butastur indicus*	*Buteo japonicus*	*Gallirallus striatus*	*Rallus indicus*	*Amaurornis akool*	*Amaurornis phoenicuru*	*Porzana fusca*	*Gallinula chloropus*	*Fulica atra*
1	0	0	1	0	0	0	0	0	0	1	0	0	0
2	0	0	0	0	0	1	0	0	1	2	0	0	0
3	2	0	0	0	0	2	0	0	0	4	0	14	0
4	0	0	0	0	0	0	0	0	0	4	0	4	0
5	0	1	0	0	1	1	0	0	0	0	0	0	0
6	0	0	0	0	0	0	0	1	0	11	1	7	0
7	0	0	0	0	0	0	0	0	0	5	0	4	0
8	0	0	0	0	0	0	0	0	0	0	0	0	0
9	0	0	0	0	0	0	0	0	0	2	0	1	0
10	0	0	0	0	0	0	0	0	0	5	0	15	0
11	0	2	0	0	0	0	0	0	0	1	0	4	0
12	0	0	0	0	0	2	0	0	0	1	0	0	0
13	0	0	0	0	0	0	0	0	0	0	0	0	0
14	0	0	0	0	0	0	0	0	0	6	0	12	0
15	0	0	0	0	0	0	0	0	0	3	0	6	0
16	0	0	0	0	0	0	0	0	0	2	0	4	0
17	0	0	0	0	0	0	0	0	0	2	0	2	0
18	0	0	0	0	0	0	0	0	0	3	0	26	2
19	0	0	0	0	0	0	0	0	0	0	0	0	0
20	0	0	0	0	0	0	0	0	0	3	0	36	0
21	0	0	0	0	0	0	0	0	0	6	2	11	0
22	1	0	0	0	0	0	0	0	0	1	0	13	0
23	0	0	0	0	0	3	0	0	1	15	0	14	0
24	1	0	0	0	0	1	0	0	0	0	0	8	0
25	0	0	0	0	0	1	0	0	0	0	0	10	0
26	0	0	0	0	0	1	2	0	0	6	0	2	0
27	0	0	0	0	0	0	0	0	1	0	0	1	0
28	0	0	0	0	0	0	0	0	0	3	0	0	0
29	0	0	0	1	0	0	0	0	0	0	0	0	0
30	0	0	0	0	0	0	0	0	0	0	0	0	0

Appendix 1

Site No.	*Turnix tanki*	*Himantopus himantopus*	*Vanellus cinereus*	*Pluvialis fulva*	*Charadrius dubius*	*Rostratula benghalensis*	*Gallinago gallinago*	*Actitis hypoleucos*	*Tringa ochropus*	*Tringa glareola*	*Tringa erythropus*	*Streptopelia orientalis*	*Spilopelia chinensis*
1	0	0	0	0	0	0	0	0	0	0	0	0	2
2	0	0	0	0	0	0	0	0	0	0	2	0	16
3	0	0	0	0	0	0	0	1	0	0	0	0	0
4	0	0	0	0	0	0	0	0	0	0	0	2	0
5	0	0	0	0	0	0	0	0	0	0	0	0	10
6	0	0	0	0	0	0	2	0	1	0	0	0	13
7	0	0	0	0	0	0	0	0	0	0	0	3	0
8	0	0	0	0	0	0	0	0	0	0	0	0	0
9	0	0	0	0	0	0	0	0	0	0	0	50	0
10	0	0	0	0	0	0	0	0	0	0	0	2	6
11	0	0	0	0	0	0	0	0	0	0	0	0	17
12	3	0	0	0	0	0	0	0	0	0	0	0	9
13	0	0	0	0	0	0	0	0	0	0	0	1	6
14	0	0	0	1	0	0	0	0	1	0	0	2	7
15	0	0	0	0	0	0	0	0	0	0	0	0	26
16	0	0	0	0	0	6	0	0	0	0	0	0	26
17	0	0	0	0	0	0	0	0	0	0	0	15	6
18	0	0	0	0	0	0	0	0	0	0	0	0	6
19	0	0	0	0	0	0	0	0	0	0	0	0	8
20	0	0	0	0	0	0	0	0	0	0	0	0	24
21	0	2	5	6	6	2	40	3	1	0	0	0	17
22	0	0	0	0	0	0	0	0	0	0	0	3	13
23	0	0	4	6	0	0	0	1	1	8	0	0	30
24	0	0	0	0	0	0	0	0	0	0	0	3	16
25	0	0	0	0	0	0	0	0	0	0	0	0	7
26	0	0	0	0	0	0	0	0	0	0	0	0	26
27	0	0	0	0	0	0	0	0	0	0	0	0	7
28	0	0	0	0	0	1	0	0	0	0	0	0	31
29	0	0	0	0	0	0	0	0	0	0	0	0	2
30	0	0	0	0	0	0	0	0	0	0	0	0	15

Appendix 1

Site No.	*Centropus sinensis*	*Centropus bengalensis*	*Clamator coromandus*	*Cacomantis merulinus*	*Hierococcyx sparverioides*	*Cuculus poliocephalus*	*Cuculus canorus*	*Glaucidium brodiei*	*Glaucidium cuculoides*	*Ninox scutulata*	*Apus pacificus*	*Apus nipalensis*
1	0	0	0	2	0	0	0	3	0	1	4	51
2	0	1	0	0	0	0	1	0	2	0	0	51
3	1	0	0	1	1	0	1	0	1	0	0	54
4	0	0	0	0	0	0	0	0	0	0	0	14
5	0	0	1	0	1	0	0	0	0	0	0	0
6	0	0	0	0	0	0	0	0	0	0	0	0
7	0	0	0	0	0	0	0	0	0	0	0	30
8	0	0	0	0	0	0	0	0	0	0	0	0
9	0	0	0	0	0	0	0	0	0	0	0	0
10	0	0	0	0	0	0	0	0	1	0	0	0
11	0	0	0	0	1	0	0	2	1	0	0	0
12	0	0	0	0	0	0	0	0	0	0	0	0
13	0	0	0	0	0	0	0	0	0	0	0	0
14	0	0	0	2	0	0	0	0	0	0	0	0
15	0	1	1	0	0	0	0	0	0	0	0	6
16	3	4	0	0	0	0	0	1	0	0	41	41
17	0	0	0	0	0	0	0	0	0	0	0	0
18	0	0	0	0	0	0	0	0	0	0	0	0
19	0	0	0	0	1	0	0	0	0	0	0	0
20	0	0	0	2	0	0	0	0	0	0	0	12
21	0	0	0	0	0	0	0	0	0	0	0	12
22	0	0	0	0	0	0	0	1	0	0	0	0
23	0	2	0	0	0	0	0	0	0	0	0	0
24	0	0	0	0	1	0	0	0	0	0	0	0
25	1	0	0	1	1	0	0	0	0	0	0	8
26	0	0	0	0	0	0	0	0	0	0	0	12
27	0	0	0	0	0	0	0	0	0	0	0	10
28	0	1	0	0	0	0	0	0	0	0	0	0
29	1	0	0	0	0	2	0	0	0	0	0	0
30	0	6	0	0	1	0	0	0	0	0	0	12

Appendix 1

Site No.	*Harpactes erythrocephalus*	*Eurystomus orientalis*	*Halcyon smyrnensis*	*Alcedo atthis*	*Alcedo hercules*	*Megaceryle lugubris*	*Merops viridis*	*Psilopogon virens*	*Psilopogon faber*	*Jynx torquilla*	*Picumnus innominatus*	*Yungipicus canicapillus*
1	0	1	0	1	0	0	0	0	0	0	0	1
2	0	0	0	6	0	0	5	0	0	0	2	0
3	0	0	0	6	0	0	6	0	0	0	0	3
4	4	4	0	1	0	0	0	1	0	0	0	4
5	0	3	0	2	0	0	0	1	0	0	0	4
6	0	0	0	0	0	0	0	0	0	0	0	0
7	0	2	0	0	0	0	0	0	0	0	0	0
8	0	0	0	0	0	0	0	0	0	1	1	0
9	0	0	0	3	0	0	0	0	0	0	0	0
10	0	0	0	0	0	0	0	0	0	0	0	0
11	1	13	0	0	0	0	0	0	1	0	0	0
12	0	0	0	0	0	0	0	3	0	0	0	4
13	1	2	0	5	0	0	0	1	0	0	0	0
14	0	0	0	0	2	0	0	0	0	0	0	1
15	1	4	0	0	0	0	0	0	0	0	0	1
16	0	6	0	0	0	0	0	0	0	0	0	0
17	2	0	0	1	0	0	6	3	0	0	0	3
18	0	3	0	1	0	0	0	8	0	0	0	1
19	0	0	0	0	0	0	0	3	0	0	0	0
20	0	0	2	10	0	0	0	0	0	0	0	0
21	0	0	3	3	0	0	0	0	0	0	0	0
22	0	9	2	2	0	0	0	0	0	0	0	0
23	0	0	1	4	0	0	11	0	0	0	0	0
24	0	4	0	3	0	0	18	1	0	1	0	0
25	0	0	0	0	0	0	0	0	0	0	0	1
26	0	4	0	1	0	0	0	0	0	0	0	2
27	0	3	0	2	0	1	0	0	0	0	0	0
28	0	2	0	6	1	0	0	1	0	0	0	6
29	0	3	0	2	0	0	6	0	0	0	0	0
30	0	0	0	0	0	0	6	0	0	0	0	1

Appendix 1

Site No.	*Picus chlorolophus*	*Picus canus*	*Blythipicus pyrrhotis*	*Micropternus brachyurus*	*Falco tinnunculus*	*Tephrodornis virgatus*	*Pericrocotus solaris*	*Pericrocotus speciosus*	*Pericrocotus divaricatus*	*Lanius schach*	*Erpornis zantholeuca*	*Pteruthius aeralatus*
1	0	0	1	1	0	0	0	0	0	23	6	0
2	0	0	2	0	0	6	64	0	0	26	0	0
3	0	0	0	2	2	0	2	0	0	21	10	0
4	0	0	0	0	0	0	0	0	0	3	0	0
5	0	0	0	0	0	1	50	51	0	2	9	0
6	0	0	0	0	0	0	0	0	0	5	0	0
7	0	0	0	0	0	0	0	21	0	0	0	0
8	0	0	0	0	0	0	0	0	0	0	0	2
9	0	0	0	0	0	0	0	0	0	0	0	0
10	0	0	0	0	0	0	0	3	0	0	0	0
11	1	0	0	0	0	14	24	15	0	0	13	1
12	1	0	3	0	0	0	0	0	0	2	0	0
13	0	0	1	0	0	0	0	3	0	4	0	0
14	0	0	0	0	0	0	0	40	0	6	0	0
15	0	0	3	0	0	0	31	0	0	5	0	0
16	0	0	0	0	0	0	2	2	0	14	0	0
17	0	0	0	0	0	20	71	18	0	8	9	0
18	0	0	0	0	0	0	3	44	0	2	11	0
19	0	0	4	0	2	0	6	0	1	4	4	0
20	0	0	0	0	0	0	0	0	0	4	0	0
21	0	0	0	0	1	0	0	0	0	11	0	0
22	0	1	0	0	0	0	0	1	0	3	0	0
23	0	0	0	0	1	0	0	0	0	16	0	0
24	0	0	0	0	0	0	2	8	0	6	4	0
25	0	0	1	0	0	0	0	0	0	5	0	0
26	0	0	0	0	1	0	0	0	0	14	0	0
27	0	0	0	0	0	0	0	1	0	9	8	0
28	0	0	0	0	0	4	8	15	0	11	0	0
29	0	0	1	0	0	0	0	40	0	1	0	0
30	0	0	0	0	0	0	1	0	0	6	0	0

Appendix 1

Site No.	*Dicrurus macrocercus*	*Dicrurus hottentottus*	*Terpsiphon e incei*	*Garrulus glandarius*	*Urocissa erythroryncha*	*Dendrocitta formosae*	*Pica serica*	*Melanochlor a sultanea*	*Parus minor*	*Machlolophu s spilonotus*	*Spizixos semitorque s*	*Pycnonotu s jocosus*
1	1	0	0	0	36	21	0	4	4	0	34	14
2	0	0	0	0	28	28	0	2	0	5	44	0
3	5	0	0	0	29	11	0	1	9	3	35	0
4	0	0	0	4	19	12	0	10	6	0	17	0
5	0	0	0	0	25	26	0	0	3	2	28	0
6	0	0	0	0	18	1	0	0	11	0	6	0
7	0	0	0	0	3	4	0	0	12	10	6	0
8	0	0	2	0	2	0	2	0	0	0	0	0
9	0	0	0	0	13	3	0	0	7	3	6	0
10	0	0	0	0	9	1	0	0	17	1	14	0
11	0	0	0	3	16	6	1	7	12	0	14	0
12	0	0	0	4	0	14	0	0	0	0	1	0
13	0	0	0	0	2	3	0	0	0	0	6	0
14	4	0	0	0	24	20	0	0	15	6	4	0
15	0	0	0	5	35	5	0	0	4	0	67	0
16	5	0	0	0	11	16	0	0	3	0	45	6
17	0	0	0	0	18	10	0	0	4	6	24	0
18	0	0	0	0	12	14	0	0	10	0	23	0
19	0	0	0	1	1	5	0	0	2	0	13	0
20	0	0	0	0	6	2	0	0	3	0	12	2
21	0	0	0	2	21	1	0	0	1	0	0	0
22	0	0	0	0	5	9	0	0	0	0	9	0
23	3	0	0	1	5	10	0	0	3	0	15	0
24	0	0	0	0	0	13	0	0	10	5	4	0
25	0	1	0	0	23	2	0	0	6	0	12	4
26	0	0	0	0	19	9	0	0	10	0	15	0
27	0	0	0	0	3	6	0	0	1	0	6	0
28	3	0	0	0	28	3	0	9	7	0	45	0
29	0	0	0	0	12	3	0	0	0	0	29	0
30	0	0	0	0	6	1	0	0	1	0	8	0

Appendix 1

Site No.	*Pycnonotus sinensis*	*Pycnonotus aurigaster*	*Hypsipetes mcclellandii*	*Hemixos castanonotus*	*Hypsipetes leucocephalus*	*Hirundo rustica*	*Delichon dasypus*	*Cecropis daurica*	*Abroscopus albogulari*	*Aegithalos concinnus*	*Phylloscopus inornatus*	*Phylloscopus proregulus*
1	33	0	8	28	52	13	14	46	6	18	0	3
2	29	0	0	3	26	0	0	30	7	11	0	0
3	25	0	0	16	11	67	0	35	6	10	10	1
4	32	0	0	3	0	30	0	6	3	0	0	0
5	10	0	0	24	6	0	0	0	6	12	0	1
6	0	0	3	0	0	16	0	50	0	0	2	0
7	17	0	0	3	0	0	40	40	0	12	0	0
8	0	0	0	0	0	0	0	0	0	0	0	12
9	1	0	0	3	0	0	0	0	0	0	0	0
10	4	0	8	10	5	0	0	0	1	0	0	0
11	7	0	0	32	3	47	0	21	2	35	0	0
12	2	0	1	2	0	0	0	5	5	70	0	0
13	0	0	0	19	38	0	0	3	0	0	0	0
14	30	0	0	6	2	0	0	22	0	11	0	2
15	18	0	0	0	0	10	0	72	1	6	0	0
16	52	0	0	0	0	20	0	25	2	23	2	0
17	32	0	1	30	51	30	0	22	4	5	0	2
18	29	0	0	12	8	0	0	0	2	1	2	3
19	0	0	0	1	0	22	0	0	0	0	0	0
20	52	0	0	0	5	34	0	16	0	0	0	2
21	10	0	0	0	0	72	0	120	1	0	0	0
22	35	0	0	10	27	23	0	11	2	0	0	2
23	41	1	0	0	0	12	12	31	1	0	0	1
24	33	0	10	32	7	0	0	0	3	20	2	14
25	0	0	0	6	0	6	0	4	0	40	1	1
26	0	0	0	1	8	22	32	52	1	0	0	0
27	22	0	5	5	20	0	0	0	0	7	1	3
28	40	0	0	6	50	9	0	36	0	15	0	1
29	46	0	0	3	18	1	0	18	2	0	0	1
30	5	0	2	3	14	2	0	2	1	0	0	0

Appendix 1

Site No.	*Phylloscopus fuscatus*	*Phylloscopus borealis*	*Prinia flaviventris*	*Prinia inornata*	*Orthotomus sutorius*	*Pomatorhinus swinhoei*	*Pomatorhinus ruficollis*	*Stachyridopsis ruficeps*	*Alcippe grotei*	*Leiothrix lutea*	*Garrulax monileger*	*Garrulax canorus*
1	2	0	0	1	1	3	4	0	16	0	0	12
2	0	0	2	6	0	0	1	3	18	0	2	8
3	0	0	2	0	0	4	3	12	19	0	6	2
4	2	0	1	1	0	0	1	9	0	0	0	0
5	3	0	0	4	0	6	2	1	14	0	0	0
6	0	0	0	4	2	0	0	0	8	0	0	0
7	0	0	0	0	1	0	0	1	0	0	0	0
8	0	0	0	0	0	0	0	0	0	0	14	0
9	0	0	0	0	0	0	0	0	1	0	3	0
10	1	0	0	0	0	2	0	0	6	0	0	0
11	0	0	0	0	0	1	0	2	16	0	0	0
12	0	0	0	0	0	3	0	5	1	0	20	0
13	1	0	0	2	0	0	3	0	3	0	0	0
14	0	0	0	5	0	0	0	4	14	0	0	12
15	0	0	1	2	4	1	1	1	6	0	0	20
16	1	0	5	13	0	3	3	3	8	0	0	14
17	0	0	1	1	0	0	5	21	21	0	0	2
18	0	0	0	12	0	0	3	7	15	0	0	0
19	0	2	1	3	0	0	2	0	19	0	0	0
20	0	0	7	5	0	0	0	0	20	0	0	0
21	1	3	1	14	0	1	0	0	0	0	0	0
22	1	0	2	0	0	2	2	1	16	6	0	0
23	0	0	2	4	0	1	1	0	0	0	0	0
24	0	0	1	8	0	1	3	5	49	1	0	1
25	0	0	1	1	0	1	0	0	1	0	0	0
26	0	0	0	1	0	0	0	0	0	0	0	0
27	0	0	0	0	0	0	1	2	53	0	0	0
28	2	0	1	4	0	2	2	5	8	0	0	0
29	1	0	1	0	0	2	3	2	10	0	0	1
30	0	1	0	0	0	2	0	5	7	0	0	0

Appendix 1

Site No.	*Pterorhinus pectoralis*	*Pterorhinus perspicillatus*	*Neosuthora davidiana*	*Psittiparus gularis*	*Yuhina torqueola*	*Zosterops simplex*	*Acridotheres cristatellus*	*Spodiopsar sericeus*	*Spodiopsar cineraceus*	*Gracupica nigricollis*	*Zoothera aurea*	*Turdus hortulorum*	*Turdus mandarinus*
1	8	2	0	0	0	0	8	0	0	0	0	0	0
2	0	24	0	0	0	0	0	0	0	0	2	0	0
3	0	0	0	0	0	10	4	0	0	0	0	0	0
4	0	0	0	0	0	10	7	0	0	0	0	0	0
5	0	0	0	0	17	12	10	0	0	0	0	0	0
6	0	0	0	0	0	0	118	0	0	0	0	0	0
7	0	4	0	0	0	0	10	0	0	4	0	0	0
8	0	0	0	0	0	0	0	0	0	4	0	0	0
9	15	4	0	0	0	6	0	0	0	0	0	0	0
10	0	0	0	0	0	0	8	0	0	0	0	0	1
11	2	0	0	0	1	0	11	0	0	0	0	0	3
12	26	0	0	0	0	0	34	0	0	0	0	0	0
13	25	5	0	0	0	0	0	0	0	2	0	0	0
14	0	4	0	0	0	0	17	9	0	6	0	0	0
15	0	2	0	0	0	40	9	0	0	10	0	0	0
16	0	20	0	0	7	0	22	0	0	32	0	0	0
17	13	0	0	30	2	10	20	0	0	0	0	0	0
18	5	17	0	0	0	0	0	0	0	0	0	0	0
19	4	0	0	0	0	0	0	0	0	0	0	0	0
20	0	0	0	0	0	0	23	80	0	10	0	0	5
21	0	0	0	0	0	0	42	0	0	12	0	0	0
22	1	6	0	0	0	0	11	0	1	0	0	0	0
23	3	9	0	0	0	15	72	12	0	10	0	0	0
24	0	0	5	0	2	0	1	0	0	0	0	3	0
25	0	0	0	0	0	0	6	0	0	0	0	0	0
26	0	6	0	0	0	0	119	0	0	21	0	0	0
27	0	0	0	0	0	5	2	0	0	0	0	0	0
28	5	3	0	0	0	0	22	0	0	2	0	0	0
29	0	1	0	0	0	0	26	0	0	4	0	0	0
30	0	0	0	0	1	0	66	0	0	0	0	0	0

Appendix 1

Site No.	*Copsychus saularis*	*Muscicapa sibirica*	*Muscicapa dauurica*	*Cyanoptila cyanomelana*	*Tarsiger cyanurus*	*Enicurus scouleri*	*Enicurus schistaceus*	*Enicurus leschenaulti*	*Myophonus caeruleus*	*Ficedula narcissina*	*Phoenicurus auroreus*	*Phoenicurus fuliginosus*
1	1	0	0	2	1	0	17	7	0	0	0	0
2	2	0	0	0	0	1	4	4	0	0	5	5
3	2	0	0	0	1	1	8	1	1	0	3	17
4	3	0	0	0	1	0	1	1	0	0	3	0
5	1	0	0	0	0	0	0	2	0	0	8	0
6	0	0	0	0	1	0	0	0	0	0	1	0
7	0	0	0	0	0	0	2	1	0	0	0	0
8	0	0	0	0	0	0	0	0	0	3	0	0
9	0	0	0	0	0	0	2	1	0	0	5	0
10	0	0	0	0	0	0	0	0	0	0	0	0
11	0	0	0	0	0	0	3	0	0	0	2	0
12	0	0	0	0	1	0	2	3	0	0	3	0
13	0	0	1	0	1	0	6	1	3	0	1	5
14	0	0	0	0	0	0	0	0	1	0	0	6
15	2	0	0	0	2	1	2	0	0	0	4	4
16	0	0	0	0	0	0	5	0	0	0	3	0
17	1	0	0	0	3	0	7	2	0	0	8	5
18	0	0	0	1	0	0	2	0	0	2	0	11
19	0	0	0	0	0	0	0	2	0	0	0	0
20	2	0	0	0	0	0	0	0	0	0	4	1
21	0	0	0	0	0	0	0	0	0	0	6	5
22	1	2	0	0	2	0	3	0	0	0	4	2
23	3	0	0	0	0	0	0	0	0	0	10	0
24	0	0	0	0	1	0	2	2	0	0	3	2
25	0	0	0	0	0	0	0	2	0	0	8	0
26	0	0	0	0	0	0	1	3	0	0	3	5
27	0	0	0	0	0	0	3	0	1	0	6	4
28	0	0	0	0	1	0	5	0	1	0	5	1
29	0	0	0	0	0	0	2	0	0	0	1	1
30	3	0	0	0	0	0	0	2	0	0	3	0

Appendix 1

Site No.	*Saxicola stejnegeri*	*Cinclus pallasii*	*Chloropsis hardwickii*	*Dicaeum ignipectus*	*Aethopyga christinae*	*Passer montanus*	*Lonchura striata*	*Lonchura punctulata*	*Motacilla tschutschensis*	*Motacilla cinerea*	*Motacilla alba*	*Anthus richardi*	*Anthus trivialis*
1	1	0	0	0	0	36	4	45	0	0	13	0	0
2	4	0	1	0	2	37	55	54	0	0	6	0	0
3	1	0	0	0	0	16	0	28	0	0	20	0	0
4	6	0	4	0	2	26	74	53	4	1	13	1	0
5	0	0	0	0	4	0	5	56	0	0	14	0	0
6	2	0	0	0	0	42	36	4	0	0	6	0	0
7	0	0	0	0	0	0	20	0	0	0	0	0	0
8	0	0	0	0	0	0	0	0	0	0	0	0	0
9	0	0	0	0	1	0	10	40	0	0	3	0	0
10	0	0	0	0	0	0	15	45	0	0	2	0	0
11	0	0	0	4	0	10	24	5	0	0	1	0	0
12	7	0	1	0	2	0	29	0	0	3	7	0	0
13	6	0	0	0	1	8	12	0	0	2	6	0	0
14	0	0	0	0	1	13	30	0	0	0	5	0	0
15	2	0	0	0	0	0	138	49	0	0	4	0	0
16	12	0	0	0	0	11	79	34	0	0	2	0	0
17	5	1	0	0	3	0	64	17	0	0	2	0	0
18	1	0	3	0	1	20	28	53	0	0	2	0	0
19	0	0	0	0	2	0	0	0	0	0	0	0	0
20	0	0	0	0	0	28	10	12	1	1	13	0	0
21	9	0	0	0	0	116	51	68	0	5	39	0	12
22	0	0	0	0	2	22	0	7	0	0	10	0	0
23	9	0	0	0	1	11	11	0	0	2	7	3	0
24	3	0	0	0	2	0	40	56	0	0	16	0	0
25	2	0	1	0	0	5	30	0	0	0	15	0	0
26	4	0	0	0	0	19	81	13	0	1	8	0	0
27	0	1	0	0	2	5	0	22	0	2	0	0	0
28	4	0	2	0	6	0	25	7	0	0	2	0	0
29	2	0	0	0	1	2	3	20	0	1	3	0	0
30	2	0	0	0	0	0	32	34	0	0	1	0	0

Appendix 1

Site No.	*Anthus hodgsoni*	*Anthus cervinus*	*Anthus rubescens*	*Fringilla montifringilla*	*Carpodacus erythrinus*	*Chloris sinica*	*Emberiza tristrami*	*Emberiza fucata*	*Emberiza pusilla*	*Emberiza chrysophrys*	*Emberiza rustica*	*Emberiza elegans*	*Emberiza rutila*
1	11	0	0	0	0	0	0	0	0	0	0	0	0
2	10	0	0	0	0	0	0	0	1	0	0	0	0
3	8	0	0	0	0	0	0	0	0	0	0	0	0
4	14	1	0	0	0	0	0	0	3	0	1	0	0
5	12	0	0	0	0	0	0	0	0	0	0	0	0
6	35	0	0	0	0	0	0	0	16	2	0	0	0
7	0	0	0	0	0	0	0	0	0	0	0	0	0
8	0	0	0	0	0	0	0	0	0	0	0	0	0
9	6	0	0	0	0	0	0	0	0	0	0	0	0
10	2	0	0	0	0	0	0	0	0	0	0	0	0
11	0	0	0	0	0	0	0	0	0	0	0	0	0
12	5	0	0	0	0	0	0	0	5	0	0	0	0
13	5	0	0	0	0	0	0	0	0	0	0	0	0
14	6	0	0	0	0	0	0	0	0	0	0	0	0
15	0	0	0	0	0	0	0	0	3	0	1	0	0
16	11	0	0	0	0	0	0	2	0	0	0	0	0
17	18	0	0	0	2	0	1	0	1	0	1	0	0
18	10	0	0	0	0	0	0	0	0	0	0	0	0
19	1	0	0	0	0	0	0	0	0	0	0	0	0
20	1	0	0	0	0	0	0	0	0	0	0	0	0
21	10	6	0	0	0	1	0	0	1	0	0	0	0
22	0	0	0	0	0	0	0	0	0	0	0	0	0
23	9	0	0	0	0	0	18	0	7	3	0	3	0
24	0	0	0	0	0	0	0	0	0	0	0	0	0
25	31	0	0	0	0	0	1	0	2	0	0	0	0
26	13	0	1	0	0	0	0	0	1	1	8	0	3
27	3	0	0	70	0	0	0	0	0	0	0	0	0
28	15	0	0	0	0	0	0	0	0	0	0	0	0
29	33	0	0	0	0	0	0	0	1	0	0	0	0
30	14	0	0	0	0	0	0	0	0	0	0	0	0

Appendix 1

Site No.	*Emberiza spodocephala*
1	0
2	0
3	5
4	10
5	0
6	17
7	0
8	0
9	0
10	0
11	0
12	10
13	0
14	0
15	0
16	12
17	7
18	0
19	0
20	6
21	20
22	0
23	40
24	5
25	21
26	22
27	0
28	5
29	6
30	6
