# Supplementary material for: Ecological drivers of avian diversity in a subtropical landscape: Effects of habitat diversity, primary productivity and anthropogenic disturbance
Source: Ecol Evol. 2022 Jul 30;12(8):e9166. doi: 10.1002/ece3.9166 (PMC9338441; doi:10.1002/ece3.9166)
Supplement: Supplementary file 2 — Appendix S2 Supporting Information [file ECE3-12-e9166-s003.pdf]

Appendix 2 Functional traits

BodyMass: adults' mean body weight (in g); **Sociality:** 0 = solitary, 1 = social; **ClutchSize:** average clutch size

DispersalRatio: mean wing length divided by cube root of mean body weight

	BodyMass	Sociality	ClutchSiz	DispersalRatio	DietPlant	DietInvertebrate	DietVertebrate	FeedingStratum
Aix galericulata	560	1	9.5	16.75	1	1	1	Ground
Anas zonorhyncha	1163	1	9.5	15.91	1	1	0	Ground
Mergus squamatus	1164	0	10	13.81	0	0	1	Ground
Arborophila gingica	253	1	6	11.62	1	1	0	Ground
Bambusicola thoracicus	271	0	8.5	19.18	1	1	0	Ground
Lophura nycthemera	1250	0	6	11.91	1	1	0	Ground
Syrmaticus ellioti	1156	1	6.5	13.78	1	1	0	Ground
Phasianus colchicus	1043.75	1	6	10.91	1	1	0	Ground
Tachybaptus ruficollis	174	1	5.5	18.92	0	0	1	Ground
Ixobrychus sinensis	94.3	0	7	18.91	0	0	1	Ground
Ixobrychus cinnamomeus	127	0	2.5	17.59	0	0	1	Ground
Nycticorax nycticorax	800	0	2	31.92	0	0	1	Ground
Butorides striata	198	1	5	19.81	0	0	1	Ground
Ardeola bacchus	306	1	3	32.01	0	0	1	Ground
Bubulcus coromandus	372	1	6	33.99	0	0	1	Ground
Ardea cinerea	1443	1	5	38.32	0	0	1	Ground
Ardea alba	873.5	1	2	36.27	0	0	1	Ground
Ardea intermedia	470.5	1	2	39.68	0	0	1	Ground
Egretta garzetta	495	0	2.5	35.1	0	0	1	Ground
Phalacrocorax carbo	2200	1	2	17.36	0	0	1	Ground
Elanus caeruleus	248	0	2	25.61	0	0	1	Air
Pernis ptilorhynchus	837.5	0	1	20.17	0	1	0	MidStorey
Aviceda leuphotes	196	0	1.5	20.35	0	0	1	Canopy
Spilornis cheela	800	0	1	38.23	0	0	1	Canopy
Ictinaetus malayensis	1027.75	0	1	51.11	0	0	1	Canopy
Accipiter trivirgatus	276	0	1.5	33.08	0	0	1	Canopy
Accipiter soloensis	161	0	3.5	39.61	0	0	1	Canopy
Accipiter virgatus	113.5	0	3.5	33.63	0	0	1	Canopy

Appendix 2 Functional traits

BodyMass: adults' mean body weight (in g); **Sociality:** 0 = solitary, 1 = social; **ClutchSize:** average clutch size

DispersalRatio: mean wing length divided by cube root of mean body weight

	BodyMass	Sociality	ClutchSiz	DispersalRatio	DietPlant	DietInvertebrate	DietVertebrate	FeedingStratum
Circus spilonotus	562.25	0	2.5	27.67	0	0	1	Canopy
Butastur indicus	402	0	3.5	21.71	0	0	1	Canopy
Buteo japonicus	878.25	0	1.5	21.15	0	0	1	Canopy
Gallirallus striatus	121	1	7	12.16	1	1	0	Ground
Rallus indicus	115.5	1	7.5	15.21	1	1	0	Ground
Amaurornis akool	137.75	1	5	13.12	0	1	0	Ground
Amaurornis phoenicurus	215.5	1	6	17.27	1	1	1	Ground
Porzana fusca	58.8	1	7	15.86	1	1	0	Ground
Gallinula chloropus	377	2	8	12.18	1	1	1	Ground
Fulica atra	775	1	9	12.86	1	1	1	Ground
Turnix tanki	47.5	0	3.5	11.29	1	1	0	Ground
Himantopus himantopus	161	1	2	21.02	0	0	1	Ground
Vanellus cinereus	270	0	2	35.57	1	1	0	Ground
Pluvialis fulva	145.5	0	2.5	31.86	0	0	1	Ground
Charadrius dubius	38.725	1	3.5	32.53	0	0	1	Ground
Rostratula benghalensis	127	1	2.5	15.21	1	1	0	Ground
Gallinago gallinago	113	0	2	15.08	0	1	0	Ground
Actitis hypoleucos	48	2	2.5	19.52	0	1	0	Ground
Tringa ochropus	79.25	0	3.5	31.12	0	1	0	Ground
Tringa glareola	67	1	2	31.06	0	1	0	Ground
Tringa erythropus	158	2	2	30.35	0	1	0	Ground
Streptopelia orientalis	233.75	1	1	31.07	1	1	0	Ground
Spilopelia chinensis	159	0	1	17.51	1	1	0	Ground
Centropus sinensis	296	0	2	31.1	0	1	0	Ground
Centropus bengalensis	159.3	0	2	31.51	0	1	0	Ground
Clamator coromandus	75.1	0	1	32.31	0	1	0	Canopy
Cacomantis merulinus	24.775	0	1	36.03	0	1	0	MidStorey
Hierococcyx sparverioides	150	0	1.5	23.69	0	1	0	MidStorey

Appendix 2 Functional traits

BodyMass: adults' mean body weight (in g); **Sociality:** 0 = solitary, 1 = social; **ClutchSize:** average clutch size

DispersalRatio: mean wing length divided by cube root of mean body weight

	BodyMass	Sociality	ClutchSiz	DispersalRatio	DietPlant	DietInvertebrate	DietVertebrate	FeedingStratum
Cuculus poliocephalus	51	0	1	21.33	0	1	0	MidStorey
Cuculus canorus	111.5	0	1	23.16	0	1	0	MidStorey
Glaucidium brodiei	56.875	0	2	11.87	0	0	1	MidStorey
Glaucidium cuculoides	205	0	2	18.01	0	0	1	MidStorey
Ninox scutulata	191	0	3	38.32	0	0	1	Air
Apus pacificus	44.425	1	1.5	29.95	0	1	0	Air
Apus nipalensis	24.575	0	3	23.29	0	1	0	Air
Harpactes erythrocephalus	80.8	0	3.5	31.8	1	1	0	Air
Eurystomus orientalis	148.3833	1	3.5	35.12	0	1	0	Air
Halcyon smyrnensis	91.4	0	6	18.17	0	0	1	Ground
Alcedo atthis	31.4	0	6	13.09	0	0	1	Ground
Alcedo hercules	85	0	5	15.97	0	0	1	Ground
Megaceryle lugubris	267.5	0	5	17.18	0	0	1	Ground
Merops viridis	35.9	0	2	32.23	0	1	0	Air
Psilopogon virens	202	0	3.5	12.98	1	1	0	Canopy
Psilopogon faber	91.35	0	3	11.16	1	1	0	Canopy
Jynx torquilla	35.86667	1	9.5	12.66	0	1	0	MidStorey
Picumnus innominatus	10.825	0	3.5	12.15	0	1	0	MidStorey
Yungipicus canicapillus	25	1	2.5	35.03	0	1	0	MidStorey
Picus chlorolophus	66.66667	1	3	30.85	0	1	0	MidStorey
Picus canus	137	1	9.5	18.08	0	1	0	MidStorey
Blythipicus pyrrhotis	146.8333	1	3	19.21	0	1	0	MidStorey
Micropternus brachyurus	88.4	1	5	17.12	0	1	0	MidStorey
Falco tinnunculus	204.75	0	2.5	20.2	0	0	1	Air
Tephrodornis virgatus	39.9	1	3	32.19	0	1	0	MidStorey
Pericrocotus solaris	14.25	0	3.5	31.51	0	1	0	MidStorey
Pericrocotus speciosus	23.3	0	3	31.12	0	1	0	Canopy
Pericrocotus divaricatus	24	0	2.5	31.61	0	1	0	MidStorey

Appendix 2 Functional traits

BodyMass: adults' mean body weight (in g); **Sociality:** 0 = solitary, 1 = social; **ClutchSize:** average clutch size

DispersalRatio: mean wing length divided by cube root of mean body weight

	BodyMass	Sociality	ClutchSiz	DispersalRatio	DietPlant	DietInvertebrate	DietVertebrate	FeedingStratum
Lanius schach	50.6	2	2.5	12.59	0	1	0	MidStorey
Erpornis zantholeuca	11.8	0	1.5	19.12	1	1	0	UnderStorey
Pteruthius aeralatus	39	1	1.5	12.8	1	1	0	MidStorey
Dicrurus macrocercus	48.5	1	3.5	38.01	0	1	0	Canopy
Dicrurus hottentottus	79.18333	0	3.5	38.11	0	1	0	Canopy
Terpsiphone incei	19.75	1	3	31.95	0	1	0	MidStorey
Garrulus glandarius	160.85	1	6.5	33.83	1	1	1	Canopy
Urocissa erythroryncha	166.25	0	2.5	32.17	1	1	1	Canopy
Dendrocitta formosae	103.75	0	2	30.8	1	1	0	Canopy
Pica serica	189	0	6.5	33.39	1	1	1	Ground
Melanochlora sultanea	37.8		6	18	1	1	0	Canopy
Parus minor	18	0	7.5	17.79	0	1	0	MidStorey
Machlolophus spilonotus	18.85	1	5	18.33	1	1	0	Canopy
Spizixos semitorques	38	0	3.5	15.77	1	1	0	Canopy
Pycnonotus jocosus	27.7	0	3	17.01	1	1	0	Canopy
Pycnonotus sinensis	36.1	1	2	16.78	1	1	0	Canopy
Pycnonotus aurigaster	45	0	1.5	17.27	1	1	0	UnderStorey
Hypsipetes mcclellandii	32.5	0	3	31.19	1	1	0	Canopy
Hemixos castanonotus	32	0	2	19.21	1	1	0	Canopy
Hypsipetes leucocephalus	46.33333	0	3	31.6	1	1	0	Canopy
Hirundo rustica	19	1	2.5	21.55	0	1	0	Air
Delichon dasypus	18	1	3	21.63	0	1	0	Air
Cecropis daurica	22.225	2	5	21.1	0	1	0	Air
Abroscopus albogularis	5	0	2.5	16.06	0	1	0	Air
Aegithalos concinnus	6.25	1	6.5	15.91	0	1	0	MidStorey
Phylloscopus inornatus	6.7625	0	5.5	17.97	0	1	0	MidStorey
Phylloscopus proregulus	6	0	5.5	18.01	0	1	0	MidStorey
Phylloscopus fuscatus	9	1	5	17.88	0	1	0	UnderStorey

Appendix 2 Functional traits

BodyMass: adults' mean body weight (in g); **Sociality:** 0 = solitary, 1 = social; **ClutchSize:** average clutch size

DispersalRatio: mean wing length divided by cube root of mean body weight

	BodyMass	Sociality	ClutchSiz	DispersalRatio	DietPlant	DietInvertebrate	DietVertebrate	FeedingStratum
Phylloscopus borealis	10	0	5.5	30.65	0	1	0	MidStorey
Prinia flaviventris	7	0	2.5	11.16	1	1	0	UnderStorey
Prinia inornata	8.475	0	5	11.19	0	1	0	UnderStorey
Orthotomus sutorius	7.5	0	2	11.65	0	1	0	MidStorey
Pomatorhinus swinhoei	54.9	1	3.5	11.83	1	1	0	UnderStorey
Pomatorhinus ruficollis	30.5	1	2	12.73	1	1	0	UnderStorey
Stachyridopsis ruficeps	10	0	2.5	12.11	1	1	0	UnderStorey
Alcippe grotei	15.1	0	2	12.6	1	1	0	MidStorey
Leiothrix lutea	21.5	1	3.5	13.73	1	1	0	MidStorey
Garrulax monileger	84	0	2	18.66	1	1	0	UnderStorey
Garrulax canorus	65.95	0	2	13.05	1	1	0	UnderStorey
Pterorhinus pectoralis	145	1	2	15.11	1	1	0	UnderStorey
Pterorhinus perspicillatus	118	1	2	12.77	1	1	0	UnderStorey
Neosuthora davidiana	159	0	3.3	15.05	1	1	0	UnderStorey
Psittiparus gularis	29	0	3	16.23	1	1	0	UnderStorey
Yuhina torqueola	11.8	0	3.5	15.15	1	1	0	MidStorey
Zosterops simplex	10.275	0	3	12.18	1	1	0	Canopy
Acridotheres cristatellus	116.5	0	2.5	16.87	1	1	0	Ground
Spodiopsar sericeus	82.25	0	6.3	18.26	1	1	0	Ground
Spodiopsar cineraceus	82.25	0	6	19.12	1	1	0	Ground
Gracupica nigricollis	157	1	5	19.35	1	1	0	Ground
Zoothera aurea	128	0	2.5	30.81	0	1	0	Ground
Turdus hortulorum	66.7	0	2	19.27	1	1	0	Ground
Turdus mandarinus	102	0	5.5	31.28	1	1	0	Ground
Copsychus saularis	36.75	0	5	17.81	0	1	0	UnderStorey
Muscicapa sibirica	13	0	2.5	31.86	0	1	0	Canopy
Muscicapa dauurica	10.375	0	5	30.68	0	1	0	Canopy
Cyanoptila cyanomelana	22.75	1	5	31.06	0	1	0	MidStorey

Appendix 2 Functional traits

BodyMass: adults' mean body weight (in g); **Sociality:** 0 = solitary, 1 = social; **ClutchSize:** average clutch size

DispersalRatio: mean wing length divided by cube root of mean body weight

	BodyMass	Sociality	ClutchSiz	DispersalRatio	DietPlant	DietInvertebrate	DietVertebrate	FeedingStratum
Tarsiger cyanurus	14	1	5.5	31.6	1	1	0	UnderStorey
Enicurus scouleri	15.9	0	3	18.72	0	1	0	Ground
Enicurus schistaceus	31.25	1	3.5	30.16	0	1	0	Ground
Enicurus leschenaulti	36.45	0	3	19.91	0	1	0	Ground
Myophonus caeruleus	160	0	2	31.76	0	1	0	Ground
Ficedula narcissina	13.1	1	2	30.11	0	1	0	Canopy
Phoenicurus auroreus	15.5	0	6.5	17.67	0	1	0	UnderStorey
Phoenicurus fuliginosus	18.8	0	2.5	16.62	0	1	0	Ground
Saxicola stejnegeri	14.7	0	6.5	16	0	1	0	MidStorey
Cinclus pallasii	77	0	2.5	11.91	0	1	0	Ground
Chloropsis hardwickii	32.75	1	3	18.73	1	1	0	Canopy
Dicaeum ignipectus	5.8	0	1.5	13.75	1	1	0	Canopy
Aethopyga christinae	5.25	0	1.5	15.25	1	0	0	Canopy
Passer montanus	22.05	1	5.5	12.7	1	1	0	Ground
Lonchura striata	12.3	1	5	11.35	1	1	0	UnderStorey
Lonchura punctulata	14	1	6	11.26	1	1	0	UnderStorey
Motacilla tschutschensis	16.55	2	5	19.36	0	1	0	Ground
Motacilla cinerea	17.2	2	5	30.76	0	1	0	Ground
Motacilla alba	24.1	1	5.5	31.76	0	1	0	Ground
Anthus richardi	27.50417	1	5	17.51	0	1	0	Ground
Anthus trivialis	23.4	1	2.5	19.5	0	1	0	Ground
Anthus hodgsoni	22.325	1	5	19.85	0	1	0	Ground
Anthus cervinus	20.81667	1	5	19.52	0	1	0	Ground
Anthus rubescens	21.18333	1	5	19.85	0	1	0	Ground
Fringilla montifringilla	22.925	0	6	31.01	1	1	0	Canopy
Carpodacus erythrinus	24.25	0	2.5	17.67	1	1	0	Canopy
Chloris sinica	19.6	1	2.5	19.55	1	1	0	UnderStorey
Emberiza tristrami	17.75	1	5.5	18.25	1	1	0	UnderStorey

Appendix 2 Functional traits

BodyMass: adults' mean body weight (in g); **Sociality:** 0 = solitary, 1 = social; **ClutchSize:** average clutch size

DispersalRatio: mean wing length divided by cube root of mean body weight

	BodyMass	Sociality	ClutchSiz	DispersalRatio	DietPlant	DietInvertebrate	DietVertebrate	FeedingStratum
Emberiza fucata	19.8	0	5	15.63	1	1	0	UnderStorey
Emberiza pusilla	14.95	0	5	17.81	1	1	0	UnderStorey
Emberiza chrysophrys	18	1	2	18.3	1	1	0	UnderStorey
Emberiza rustica	20.1	0	2.5	18.58	1	1	0	UnderStorey
Emberiza elegans	17.175	0	6	17.99	1	1	0	UnderStorey
Emberiza rutila	17.375	0	2.5	17.11	1	1	0	UnderStorey
Emberiza spodocephala	17.725	0	2.5	15.19	1	1	0	UnderStorey
