# Supplementary material for: Ecological drivers of avian diversity in a subtropical landscape: Effects of habitat diversity, primary productivity and anthropogenic disturbance
Source: Ecol Evol. 2022 Jul 30;12(8):e9166. doi: 10.1002/ece3.9166 (PMC9338441; doi:10.1002/ece3.9166)
Supplement: Supplementary file 3 — Appendix S3 Supporting Information [file ECE3-12-e9166-s004.pdf]

Appendix 3 Diversity Indices

sesPhyloMPD: standardized effects of phylogenetic mean pairwise distance
sesPhyloMNTD: standardized effects of phylogenetic mean nearest taxon distance
sesFunctMPD: standardized effects of functional mean pairwise distance
sesFunctMNTD: standardized effects of functional mean nearest taxon distance

Site No.	Richness	Rarefied	sesPhyloMPD	sesPhyloMNTD	sesFunctMPD	sesFunctMNTD
Site1	56	23.03	-4.28	-2.97	-4.18	-2.32
Site2	62	22.70	-3.57	-0.87	-2.21	-1.11
Site3	67	25.05	-3.17	-0.58	-2.71	-2.17
Site4	56	22.68	-3.36	-2.09	-1.32	-1.71
Site5	51	21.77	-3.40	-1.84	-2.10	-1.10
Site6	38	16.93	-2.28	-0.25	-0.78	-1.72
Site7	27	16.69	-4.04	-1.13	-2.47	-1.59
Site8	11	12.00	0.41	1.75	0.10	0.11
Site9	29	17.05	-1.06	-1.54	-2.47	-2.08
Site10	28	17.82	-2.60	-2.75	-3.62	-1.87
Site11	52	23.37	-4.26	-2.41	-2.19	-2.03
Site12	42	19.45	-0.71	-1.54	-3.21	-1.91
Site13	42	22.21	-1.02	-2.11	-2.64	-1.52
Site14	42	23.06	-2.89	-0.50	-2.78	-1.13
Site15	50	17.87	-2.87	-1.55	-3.86	-2.93
Site16	53	23.55	-3.91	-3.00	-2.52	-3.11
Site17	61	24.11	-4.30	-2.53	-5.95	-3.73
Site18	50	23.40	-2.26	-2.35	-1.49	-1.53
Site19	28	18.95	-2.68	0.19	-3.16	-0.76
Site20	43	20.17	-2.55	-1.83	-0.83	-2.16
Site21	55	20.07	-2.93	-2.70	0.62	-0.76
Site22	57	25.31	-2.52	-1.90	0.01	-0.36
Site23	60	25.08	-1.06	-1.52	-0.93	-0.77
Site24	57	22.96	-2.98	-2.38	-2.94	-1.82
Site25	45	20.39	-0.83	0.14	-1.32	-1.58
Site26	46	19.53	-3.33	-2.21	-1.63	-2.01
Site27	44	19.86	-1.89	-0.40	-1.16	-1.74
Site28	60	23.85	-3.38	-1.41	-2.91	-2.69
Site29	50	20.03	-1.53	-0.24	-1.96	-2.21
Site30	37	18.08	-2.85	0.21	-4.09	-2.57
