# Supplementary material for: Ecological drivers of avian diversity in a subtropical landscape: Effects of habitat diversity, primary productivity and anthropogenic disturbance
Source: Ecol Evol. 2022 Jul 30;12(8):e9166. doi: 10.1002/ece3.9166 (PMC9338441; doi:10.1002/ece3.9166)
Supplement: Supplementary file 4 — Appendix S4 Supporting Information [file ECE3-12-e9166-s002.pdf]

Appendix 4 Ecological Factors

Conservation: whether the site is located in the natural reserve; **HPD:** human population density

BirdingHistory: number of years since the launch of bird watching tourism

LargestForest: area of largest forest patch

Sites	Longtitude	Latitude	Elevation (m)	Conservation	NDVI	HPD	BirdingHistory	HabitatDiversity	LargestForest
Site1	117.51	26.55	988	No	0.81	55.91	0.00	1.07	132.53
Site2	117.52	26.53	825	No	0.89	58.17	0.00	0.92	142.76
Site3	117.56	26.48	816	No	0.85	117.65	0.00	1.03	165.37
Site4	117.54	26.46	812	No	0.83	56.56	0.00	1.20	147.18
Site5	117.53	26.42	804	No	0.87	56.66	0.00	1.06	145.69
Site6	117.48	26.36	783	Yes	0.82	56.86	7.00	1.46	50.59
Site7	117.46	26.37	781	Yes	0.90	58.90	7.00	0.74	149.50
Site8	117.47	26.37	756	Yes	0.90	56.74	7.00	0.88	68.22
Site9	117.48	26.37	749	Yes	0.81	56.76	0.00	1.16	57.17
Site10	117.48	26.37	649	Yes	0.81	56.76	7.00	1.22	57.17
Site11	117.35	26.43	635	No	0.90	70.46	2.00	0.94	86.67
Site12	117.34	26.42	589	Yes	0.90	55.88	4.00	1.08	108.00
Site13	117.41	26.39	569	No	0.89	56.41	0.00	0.91	182.95
Site14	117.31	26.33	552	No	0.89	56.45	0.00	1.21	125.12
Site15	117.30	26.37	533	No	0.89	58.38	4.00	1.16	94.29
Site16	117.28	26.34	504	No	0.84	56.72	0.00	1.37	93.95
Site17	117.14	26.29	483	No	0.90	56.57	4.00	0.99	151.32
Site18	117.13	26.31	427	No	0.85	56.49	2.00	1.19	90.03
Site19	117.20	26.32	403	No	0.90	56.45	0.00	0.66	209.29
Site20	117.19	26.36	379	No	0.49	75.00	7.00	1.58	46.99
Site21	117.22	26.36	367	No	0.68	80.94	0.00	1.52	36.47
Site22	117.11	26.53	364	Yes	0.90	55.73	3.00	1.20	125.46
Site23	117.15	26.48	364	No	0.85	70.52	3.00	1.34	102.00
Site24	117.24	26.17	359	No	0.84	57.35	0.00	1.30	117.38
Site25	117.25	26.26	343	No	0.90	56.81	3.00	1.16	142.05
Site26	117.00	26.59	333	Yes	0.90	55.66	0.00	1.34	75.76

Site27	117.05	26.56	329	Yes	0.88	70.29	0.00	1.20	139.05
Site28	117.00	26.50	326	No	0.88	70.62	4.00	1.22	118.49
Site29	117.01	26.55	301	No	0.86	55.72	0.00	1.25	134.99
Site30	116.87	26.53	252	Yes	0.89	58.28	4.00	1.17	109.75
